# Functional dichotomy of Vδ2 γδ T cells in chronic hepatitis C virus infections: role in cytotoxicity but not for IFN-γ production

**DOI:** 10.1038/srep26296

**Published:** 2016-05-19

**Authors:** Wenwei Yin, Shiwen Tong, Qiongfang Zhang, Jianying shao, Qian Liu, Hong Peng, Huaidong Hu, Mingli Peng, Peng Hu, Hong Ren, Zhigang Tian, Dazhi Zhang

**Affiliations:** 1 Key Laboratory of Molecular Biology for Infectious Diseases (Ministry of Education), Institute for Viral Hepatitis, Department of Infectious Diseases, The Second Affiliated Hospital, Chongqing Medical University, Chongqing, P. R. China; 2 Institute of Immunology, School of Life Sciences, University of Science and Technology of China, Hefei, China

## Abstract

Vδ2 γδ (Vδ2) T cells, a major human γδ T cell subset, exhibit broad anti-tumor and anti-infective activity; however, their precise role in chronic hepatitis C virus (HCV) infections remains unclear. In this study, we analyzed the phenotype and function of Vδ2 T cells in 43 HCV-infected patients compared to 39 healthy controls (HCs). Vδ2 T cells from HCV-infected patients were activated and differentiated into effector cells. Vδ2 T cells in patients expressed significantly higher levels of natural killer (NK) cell markers CD56 and CD16 than in HCs, acquiring cytotoxic NK-like phenotype. The Vδ2 T cell phenotype was associated with increased cytolytic effector molecules expression in HCV-infected patients with elevated serum ALT levels. Surprisingly, Vδ2 T cells in patients had a markedly impaired capacity to produce IFN-γ. Further *in vitro* and *in vivo* analysis showed that interferon-α, which was induced during HCV infection, caused Vδ2 T cell function bias toward cytotoxicity. These results suggest a functional dichotomy for Vδ2 T cells in chronic HCV infections: a role in cytotoxicity but not for IFN-γ production, which may contribute to both the liver inflammation and HCV persistence.

Hepatitis C virus (HCV) causes persistent infection in more than 70% of cases. HCV infection is closely associated with chronic liver inflammation, which may progress to fibrosis, cirrhosis, or hepatocarcinoma. In general, HCV is not directly cytopathic for infected hepatocytes, and liver injury and disease progression are immune mediated^1^. The host immune response induced by persistent HCV infection contributes not only to viral control but also to liver injury^1^,^2^. Chronic HCV infection is characterized by severe immune dysregulation resulting in liver injury and viral persistence^3^. As to date, the reason why immune system leads to liver injury but can not eradicate HCV is not completely understood. Whereas previous studies have paid much attention to the characteristics and role of CD8 + T cells, CD4 + T cells, and NK cells in chronic HCV infections^4^,^5^, relatively little work has been done on the features of γδ T cells in the context of HCV persistence.

In humans, γδ T cells represent 1–5% of the circulating T cells in blood, with the majority (50–95%) expressing a Vγ9Vδ2 TCR (hereafter referred as Vδ2 T cells) that serves as an important innate immune component against microbial agents and tumors^6^,^7^. Cells in this subset reacts in a major histocompatibility complex (MHC)-unrestricted manner to a set of low m.w. nonpeptide phosphoantigens such as the mevalonate pathway-derived isopentenyl pyrophosphate (IPP) or synthetic phosphoantigen such as bromohydrin pyrophosphate(BrHPP)^8^,^9^. Once activated, Vδ2 T cells rapidly secrete high levels of cytokines such as IFN-γ and kill target cells^10^. Vδ2 T cells have been shown to exert a broad antiviral activity against different viruses such as human immunodeficiency virus (HIV), influenza A (fluA) and could also contribute to the pathology associated with these infections^11^,^12^,^13^. Our group previously reported that Vδ2 T cells were involved in immune response to hepatitis B virus (HBV)^14^,^15^,^16^, another virus that targets liver. Recently, emerging evidence has indicated that Vδ2 T cells might be implicated in HCV infection^17^,^18^. Patients with chronic HCV infection show elevated intrahepatic γδ T cells and that γδ T cells have strong cytotoxic activity against hepatocytes, suggesting a pathogenic role for γδ T cells in HCV infection^19^. Anti-HCV potential of Vδ2 T cells is also expected. *In vitro* activation of Vδ2 T cells by nonpeptidic antigen inhibits HCV replication and the antiviral activity is mainly mediated by the release of IFN-γ^20^. Although these studies have partially defined the role of Vδ2 T cells in human HCV infection, the detailed characteristics of Vδ2 T cells during chronic HCV infection need further investigation.

In the present study, we analyzed the phenotype and function of Vδ2 T cells in patients with chronic HCV infection. We observed that Vδ2 T cells showed an activated/effector phenotype in HCV-infected patients; in contrast to their upregulated cytolytic enzymes expression and maintenance of degranulation, Vδ2 T cells in patients had a markedly impaired capacity to produce IFN-γ. This polarized phenotype was associated with liver injury and was induced by exposure to IFN-α.

## Results

### Vδ2 T cells are activated and differentiate into effector cells in HCV-infected patients

To explore γδ T cell effector potential in the context of chronic HCV infection, we first analyzed the frequencies of peripheral γδ T cells and Vδ1 and Vδ2 subsets in 43 HCV-infected patients compared to 39 HCs (Supplementary Table S1). No significant differences in the frequency of γδ T cells and Vδ1 and Vδ2 subsets were observed between HCV-infected patients and HCs (Fig. S1A,B). However, the absolute number of circulating Vδ2 T cells showed significant positive correlation with serum ALT levels (r = 0.4049; *P* = 0.0073; Fig. S1E), but not with serum HCV RNA loads (r = 0.0133; *P* = 0.9334; Fig. S1F). No correlation was found between the number of Vδ1 T cells with either serum ALT levels or HCV RNA loads (r = 0.0429; *P* = 0.7875 and r = 0.0474; *P* = 0.7656; Fig. S1C,D). Therefore, we focus our following studies on Vδ2 T cells.

**Figure 1:**
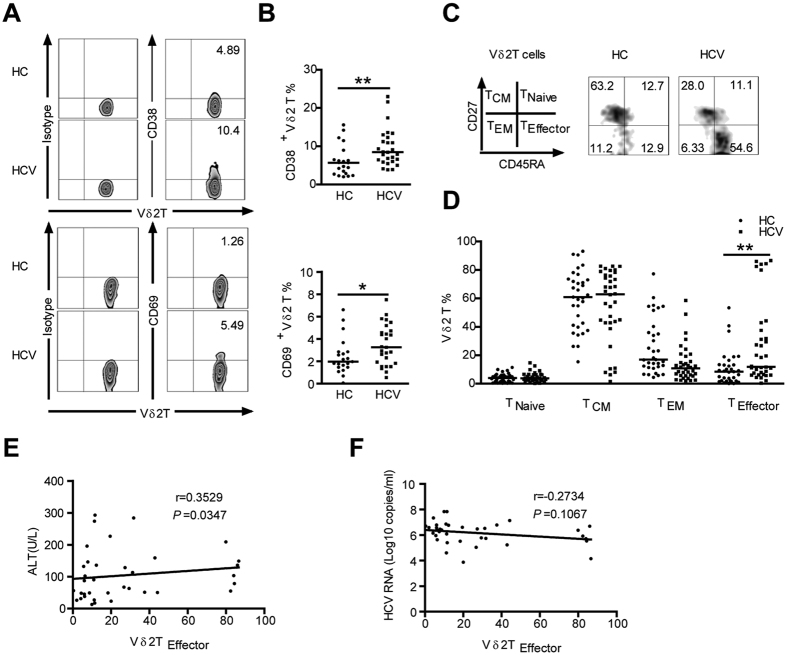
Vδ2 T cells in HCV-infected patients show an activated terminally differentiated effector phenotype. (**A**) Representative flow cytometry panels depict the expression of the activation markers CD38 and CD69 on Vδ2 T Cells from HCs and HCV-infected patients. (**B**) Frequency of Vδ2 T cells expressing CD38 and CD69 in HCs and HCV-infected patients. n = 21 and 27 for HC and HCV, respectively. (**C**) Distribution of Vδ2 T cell subsets in representative individuals from HCs and HCV-infected patients. Differentiation was analyzed by monitoring CD27 and CD45RA expression. (**D**) Vδ2 T cell differentiation profile from HCs and HCV-infected patients. n = 33 and 36 for HC and HCV, respectively. Horizontal lines indicate the median. (**E,F**) Correlation analysis of the percentages of Vδ2 T_Effector_ cells and the serum ALT levels (**E**) or HCV RNA loads (**F**) in HCV-infected patients. n = 36. *p < 0.05, **p < 0.01.

We observed that peripheral Vδ2 T cells from patients expressed significantly higher levels of activation marker CD38 and CD69 than those from HCs (Fig. 1A,B), suggesting that Vδ2 T Cells are activated *in vivo* in HCV-infected patients.

Vδ2 T cells are heterogeneous and comprise distinct populations that can be distinguished based on surface marker CD27 and CD45RA expression: naive (T_Naive_; CD45RA + CD27+) and central memory (T_CM_; CD45RA-CD27+) cells that home to secondary lymphoid organs, but lack immediate effector functions, while effector memory (T_EM_; CD45RA-CD27-) and terminally differentiated effector (T_Effector_; CD45RA + CD27-) cells that home to sites of inflammation where they display immediate effector functions such as cytokine production and cytotoxic activity^21^. Vδ2 T cells from HCV-infected patients were found to contain more effector cells (CD45RA + CD27-) than those from HCs (Fig. 1C,D), suggesting that chronic HCV infection induced V**δ**2 T-cell differentiation into effector cells. In addition, a significant positive correlation was observed between the frequency of Vδ2 T_Effector_ cells and serum ALT levels (r = 0.3529; *P* = 0.0347; Fig. 1E), interestingly, no correlation was found with serum HCV RNA loads in patients (r = −0.2734; *P* = 0.1067; Fig. 1F). These data suggest that Vδ2 T cells displayed an activated/effector phenotype and were implicated in causing liver injury in HCV-infected patients.

### Vδ2 T cells from HCV-infected patients acquire cytotoxic NK-like phenotype

**Figure 2:**
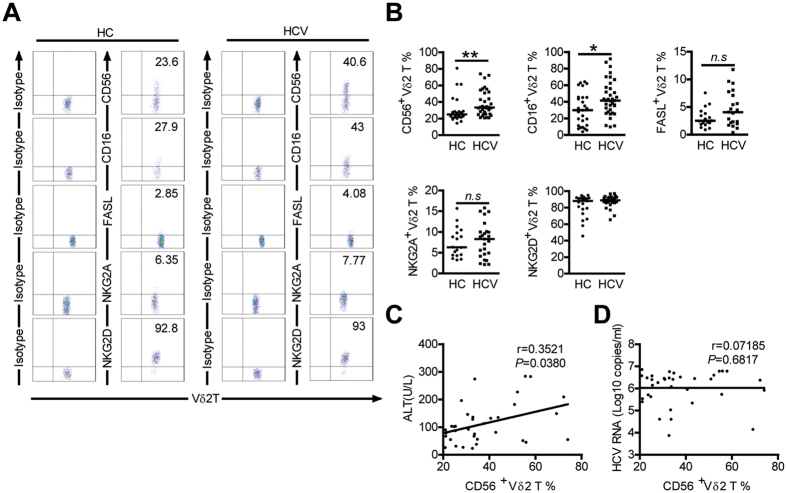
Comparison of the expression of NK cell-associated antigens on Vδ2 T cells in HCs and HCV-infected patients. (**A**) Representative dot plots show the expression of CD56, CD16, FasL,NKG2A and NKG2D on Vδ2 T cells from HCs and HCV-infected patients. (**B**) Frequency of Vδ2 T cells displaying the indicated surface markers in HCs and HCV-infected patients. CD56, n = 25 and 35, respectively; CD16, n = 27 and 36, respectively; FasL, n = 19 and 21, respectively; NKG2A n = 18 and 25, respectively; NKG2D, n = 21 and 32, respectively. Horizontal lines indicate the median. (**C,D**) Correlation analysis of the percentages of CD56 + Vδ2 T cells and the serum ALT levels (**C**) or HCV RNA loads (**D**) in HCV-infected patients. n = 35. *p < 0.05, **p < 0.01.

It has been reported that Vδ2 T cells express several natural killer (NK) cell-associated antigens with relevance for their activity^22^. We found that the Vδ2 T cells in HCV-infected patients were higher than controls for the percentage of cells expressing CD56 or CD16, NK-cell markers associated with cytotoxic effector function in this cell type (Fig. 2A,B)^23^,^24^. No significant differences in expression of NK-cell receptors FasL, NKG2A and NKG2D on Vδ2 T cells was observed between patients and HCs (Fig. 2A,B). Further analysis showed that the frequency of CD56 + Vδ2 T cells in patients correlated positively with serum ALT levels (r = 0.3521; *P* = 0.0380; Fig. 2C) but had no relationship with serum HCV RNA loads (r = 0.07185; *P* = 0.6817; Fig. 2D), arguing for their active role in disease progression. Vδ2 T cells expressing CD56 or CD16 were previously shown to be more cytotoxic^23^,^24^, indicating Vδ2 T cells are potentially prone to cytotoxicity *in vivo* in HCV-infected patients.

### Enhanced cytolytic effector molecules production by Vδ2 T Cells associates with liver damage in HCV-infected patients

**Figure 3:**
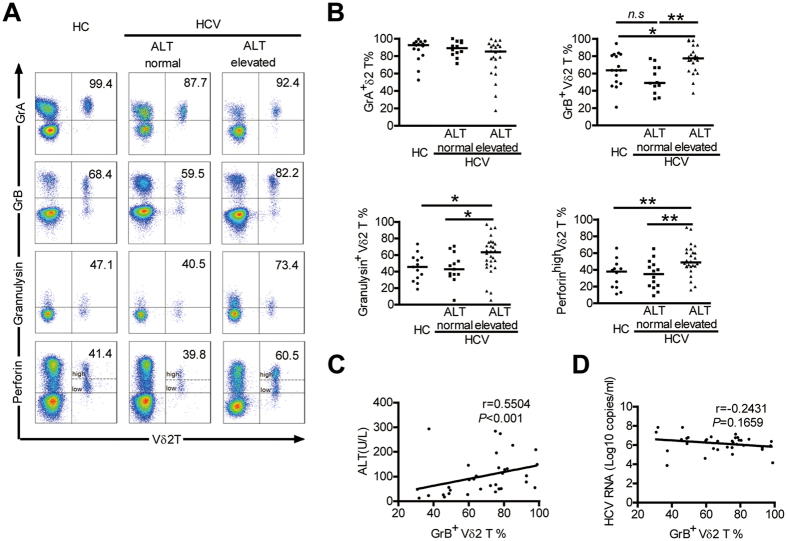
Enhanced cytolytic enzymes production by Vδ2 T Cells associates with liver damage in HCV-infected patients. (**A**) Representative dot plots depict the expression of GrA, GrB, Granulysin and perforin on Vδ2 T cells from HCV-infected patients with elevated ALT levels and from those with normal ALT levels and HCs. (**B**) Frequency of Vδ2 T cells expressing GrA, GrB, Granulysin (gated on CD3 + T cells) and perforin (gated on lymphocytes) in each group. GrA, n = 15 and 12 and 22, respectively; GrB, n = 16 and 12 and 22, respectively; Granulysin, n = 13and 13 and 28, respectively; perforin, n = 12 and 14 and 26, respectively. Horizontal lines indicate the median. (**C,D**) Correlation analysis of the percentages of GrB + Vδ2 T cells and the serum ALT levels (**C**) or HCV RNA loads (**D**) in HCV-infected patients. n = 34. *p < 0.05, **p < 0.01.

In order to determine whether the cytotoxic phenotype of Vδ2 T cellscorrelates with their cytotoxic functions, we further investigated the concentration of cytolytic effector molecules (GrA, GrB, granulysin, and perforin) that are responsible for Vδ2 T Cell cytotoxicity. Our data showed that GrB, granulysin, and perforin production by Vδ2 T cells were substantially increased in HCV-infected patients with elevated ALT levels, compared with Vδ2 T cells from HCV-infected patients with normal ALT levels and HCs (Fig. 3A,B). Moreover, the percentage of GrB + Vδ2 T cells had a significant positive correlation with serum ALT levels (r = 0.5504; *P < *0.001; Fig. 3C), but showed no correlation with serum HCV RNA load (r = −0.2431; *P* = 0.1659; Fig. 3D). These data revealed that high levels of cytolytic enzymes expression by Vδ2 T Cells were closely associated with liver damage in HCV-infected patients.

### Vδ2 T Cells display impaired IFN-γ production in patients with chronic HCV

**Figure 4:**
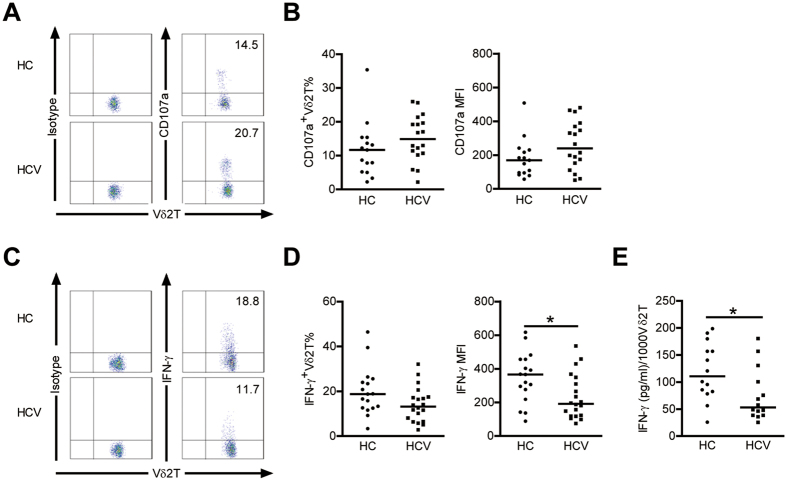
Vδ2 T cells display enhanced degranulation but decreased IFN-γ production in patients with chronic HCV. (**A**) Representative dot plots depict CD107a expression on Vδ2 T cells from HCs and HCV-infected patients. (**B**) Frequency and mean fluorescence intensity (MFI) of Vδ2 T cells expressing CD107a in HCs and HCV-infected patients. n = 15 and 18 for HC and HCV, respectively. (**C**) Representative dot plots depict IFN-γ expression on Vδ2 T cells from HCs and HCV-infected patients. (**D**) Frequency and mean fluorescence intensity (MFI) of Vδ2 T cells expressing IFN-γ in HCs and HCV-infected patients. n = 17 and 20 for HC and HCV, respectively. (**E**) A quantitative analysis of IFN-γ produced by Vδ2 T cells from HCs and HCV-infected patients was performed by ELISA assay. Results (pg/ml of IFN-γ) were normalized for 1000 Vδ2 T cells. n = 14 for each group. Horizontal lines indicate the median. *p < 0.05.

To further assess Vδ2 T cell cytolytic potential, we evaluated the ability of Vδ2 T cells to produce CD107a, a marker of degranulation. Upon zoledronate stimulation, the expression of CD107a was enhanced, although not statistically significant, in Vδ2 T cells from HCV-infected patients compared to HCs (Fig. 4A,B), suggesting that Vδ2 T Cells in HCV infected patients exhibit normal or enhanced degranulation.

IFN-γ is a key cytokine which has direct non-cytolytic antiviral effects on HCV replication, and Vδ2 T cells have been proposed as an early source for this cytokine^20^,^25^; therefore, we evaluated IFN-γ production. Unexpectedly, the frequency of IFN-γ producing Vδ2 T cells in HCV-infected patients did not increase and the corresponding mean fluorescence intensity showed a significant decrease (Fig. 4C,D). The enzyme immunoassays confirmed that IFN-γ production by Vδ2 T cells decreased in HCV infected patients (Fig. 4E). Interestingly, we detected very low levels of IL-17 expression on Vδ2 T cells with stimulation by PMA-ionomycin or zoledronate in HCs and HCV-infected patients (data not shown). So, in contrast to the observed increased cytolytic enzymes production and degranuation, IFN-γ-producing capacity of Vδ2 T cells was severely impaired in HCV infected patients.

### Hepatic Vδ2 T cells exhibit higher levels of activation and cytotoxic markers than those in peripheral compartments in HCV-infected patients

**Figure 5:**
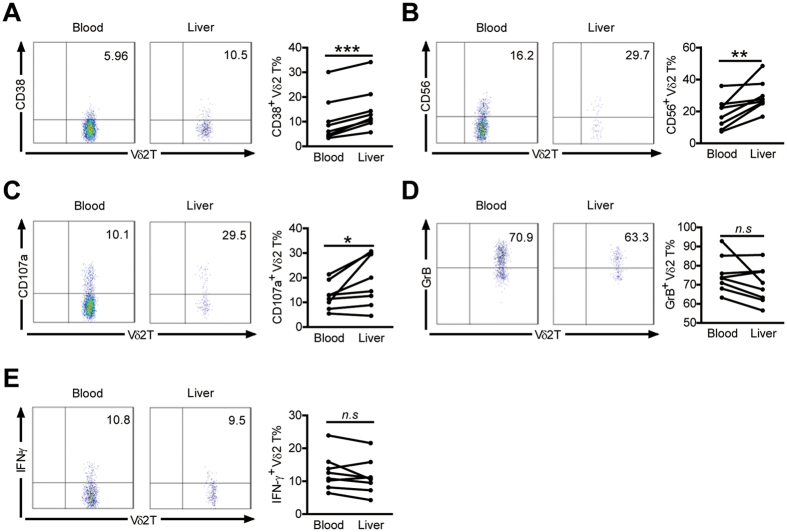
Expression of activation and cytotoxic markers by hepatic Vδ2 T cells is higher than those in peripheral blood in HCV-infected patients. (**A,B,D**) The proportions of Vδ2 T cells expressing CD38 (**A**), CD56 (**B**), GrB (**D**) were compared in HCV-infected patients with paired liver and blood samples. n = 8 for each group. (**C,E**) The proportions of Vδ2 T cells producing CD107a (**C**) and IFN-γ (**E**) upon zoledronate stimulation are also shown. n = 8 for each group. *p < 0.05, **p < 0.01, ***p < 0.001.

We compared the activation status and functions of the hepatic and peripheral Vδ2 T cell compartments in HCV-infected patients with paired peripheral and intrahepatic samples (Supplementary Table S2). Hepatic Vδ2 T cells expressed higher levels of activation marker CD38 and cytotoxic marker CD56 in comparison with peripheral Vδ2 T compartments (Fig. 5A,B). Hepatic Vδ2 T cells also exhibited significantly higher expression of surface degranulation marker CD107a but slightly reduced expression of GrB than peripheral Vδ2 T cells, which most likely can be attributed to the increased release of cytolytic granules from hepatic Vδ2 T cells as a consequence of repeated contact with HCV-infected cells and subsequent degranulation (Fig. 5C,D). However, IFN-γ production from hepatic Vδ2 T cells was not enhanced in comparison with peripheral Vδ2 T cells (Fig. 5E). These data indicate that hepatic Vδ2 T cells displayed higher levels of activation and cytotoxic functions than peripheral Vδ2 T cells in HCV-infected patients.

### *In vitro* IFN-α stimulation enhances Vδ2 T cell activation and cytolytic activity but not IFN-γ production

**Figure 6:**
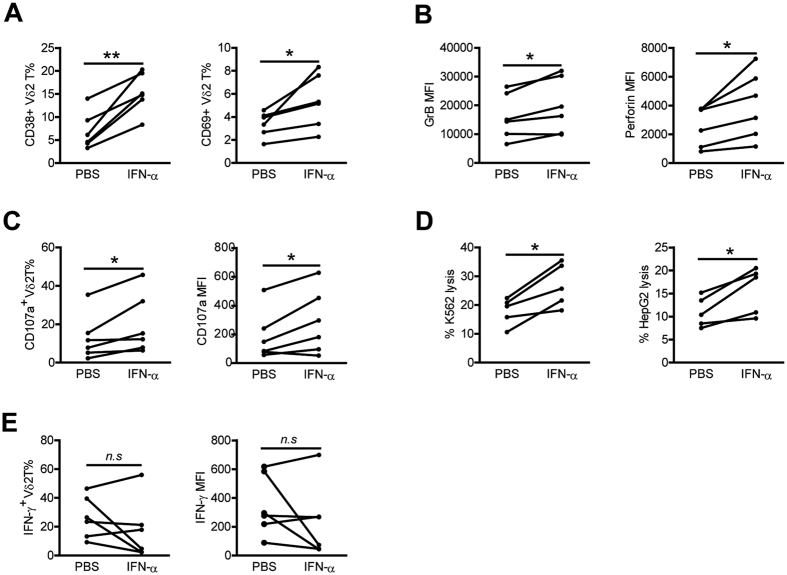
Vδ2 T cells of HCs are activated and upregulate cytolytic activity but not IFN-γ production after *in vitro* exposure to IFN-α. (**A–C,E**) PBMCs from HCs were preincubated with or without IFN-α for 24 h. (**A,B**) Expression of activation markers CD38 and CD69 (**A**), and cytolytic enzymes GrB and perforin (**B**) on Vδ2 T cells was assessed by flow cytometry. (**C,E**) Expression of CD107a (**C**) and IFN-γ (**E**) on Vδ2 T cells upon zoledronate stimulation was analyzed by flow cytometry. n = 6 for each group. (**D**) Percentages of K562 and HepG2 lysis byVδ2T cell lines preincubated with or without IFN-α for 24 h. n = 5 for each group. *p < 0.05, **p < 0.01.

HCV infection triggers rapid and sustained production of type I IFN and it has been reported that chronically HCV-infected patients show levels of biologically active IFN-α in the serum before initiation of therapy^26^,^27^. To assess whether IFN-α induced the polarized Vδ2 T cell cytolytic phenotype in HCV-infected patients, PBMCs from HCs were stimulated *in vitro* with IFN-α. Vδ2 T cells from HCs were shown to exhibit significant up-regulation in the expression of activation markers CD38 and CD69 upon IFN-α stimulation (Fig. 6A). Activation of Vδ2 T cells by IFN-α also increased Vδ2 T-cell cytolytic enzymes GrB and perforin expression (Fig. 6B) and degranulation (Fig. 6C). Furthermore, pretreatment with IFN-α markedly augmented cytotoxicity of Vδ2 T cells against K562 and HepG2 target cells (Fig. 6D). However, IFN-α stimulaiton did not significantly increase the frequency of IFN-γ–producing Vδ2 T cells and the corresponding mean fluorescence intensity (Fig. 6E). So, IFN-α was more potent for enhancing Vδ2 T cell cytotoxic activity. We also observed similar effects with IFN-α on Vδ2 T cells from HCV-infected patients (Fig. S2A–D). These data suggest that *in vitro* exposure of Vδ2 T cells to IFN-α caused Vδ2 T cells to become functionally biased towards cytolytic phenotype, but that lacked an increase in IFN-γ.

**Figure 7:**
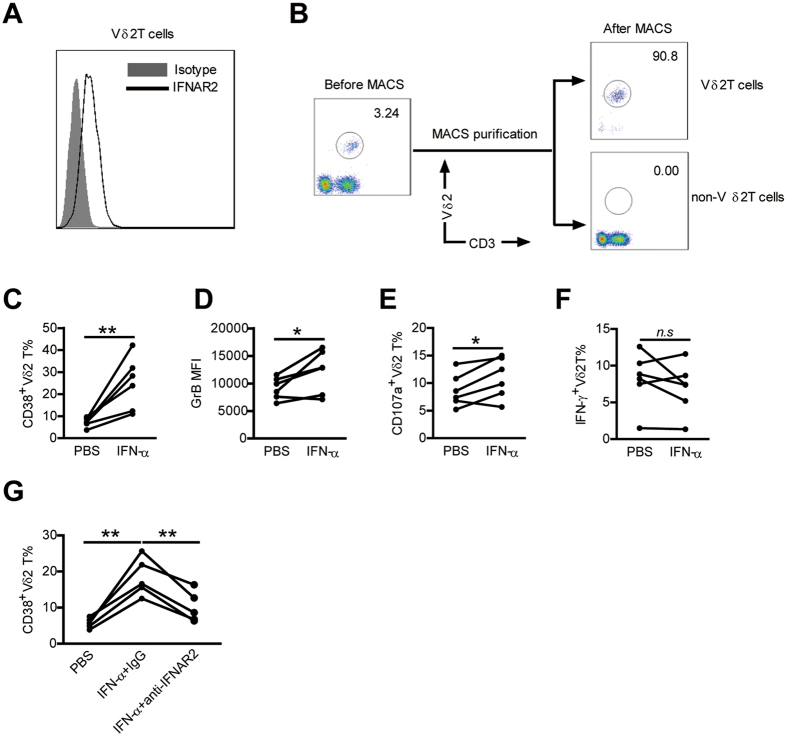
IFN-α could directly modulate Vδ2 T-cell phenotype and function. (**A**) PBMCs from HCs were stained with an unlabeled primary antibody against IFNAR2 or an isotype-matched control antibody (mouse IgG2a), and then by a phycoerythrin (PE)-conjugated anti- mouse IgG2a secondary antibody. IFNAR2 expression on Vδ2 T cells was analyzed. A representative staining profile is shown. (**B**) PBMCs of healthy blood donors were separated for Vδ2 T cells and non- Vδ2 T cells using magnetic beads. Purified cells were stained with anti- Vδ2 T mAb and anti-CD3 mAb and analyzed by flow cytometry. The representative dot plots from six experiments are shown. (**C–F**) Purified Vδ2 T cells were preincubated with or without IFN-α for 24 h. (**C,D**) Expression of activation markers CD38 (**C**), and cytolytic enzymes GrB (**D**) on Vδ2 T cells was assessed by flow cytometry. (**E,F**) Expression of CD107a (**E**) and IFN-γ (**F**) on Vδ2 T cells upon zoledronate stimulation was analyzed by flow cytometry. n = 6 for each group. (**G**) Purified Vδ2 T cells were cultured in medium containing PBS or 100 U ⁄ml IFN-α, a blocking antibody against type I IFN receptor (anti-IFNAR2, 5 μg ⁄ml) or an isotype control antibody was added to the medium. After 24 hr of culture, the expression of CD38 on Vδ2 T cells was assessed. n = 5. *p < 0.05, **p < 0.01.

As Vδ2 T cells expressed type I IFN-receptor (Fig. 7A), we further determined whether IFN-α could act directly on Vδ2 T cells or function indirectly by affecting other cells, especially monocytes. We purified Vδ2 T cells from PBMCs and stimulated those cells with IFN-α, then cultured those cells with the remaining untreated lymphocyte populations (non-Vδ2 T cells) in the presence of zoledronate (Fig. 7B). The results showed that direct stimulation of Vδ2 T cells by IFN-α increased the expression of CD38 and GrB (Fig. 7C,D). Moreover, Vδ2 T cells pretreated by IFN-α produced more CD107a but not IFN-γ upon zoledronate stimulation in comparison with those pretreated by PBS (Fig. 7E,F). The addition of a blocking Ab against type I IFN receptor markedly reduced the CD38 upregulation on Vδ2 T cells by IFN-α (Fig. 7G), highlighting the importance of type I IFN signaling in our experimental system. These data suggest that IFN-α could directly modulate Vδ2 T-cell phenotype and function.

### *In vivo* IFN-α administration further decreases Vδ2 T cell IFN-γ production in HCV-infected patients

**Figure 8:**
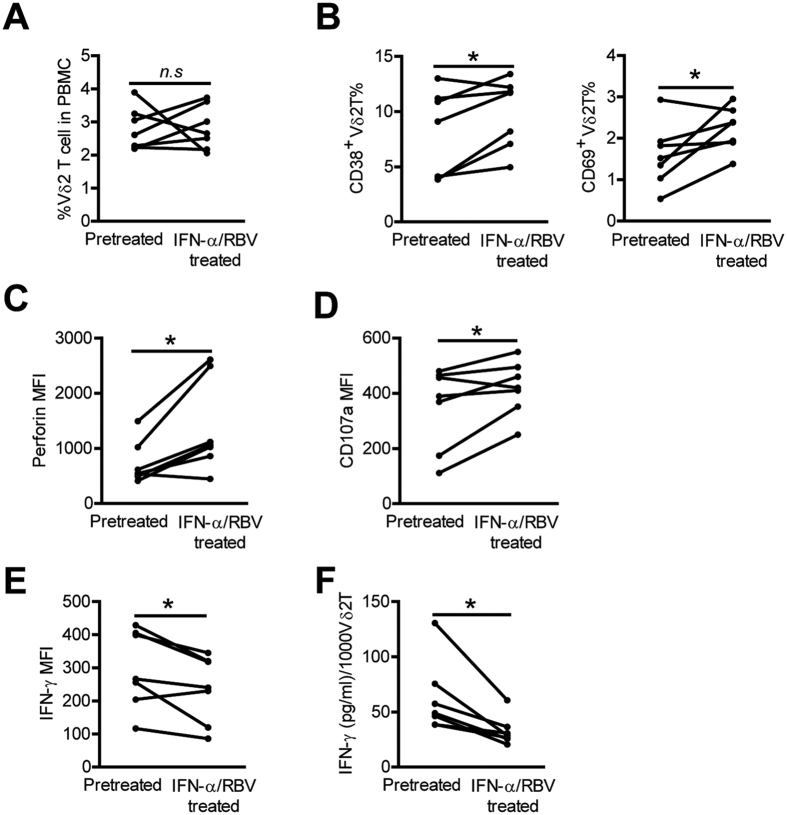
*In vivo* IFN-α administration further enhances Vδ2 T cell activation and cytolytic activity but decreased Vδ2 T cell IFN-γ production in HCV-infected patients. (**A–C**) The frequency of Vδ2 T cells (**A**), and expression of activation markers CD38 and CD69 (**B**), and cytolytic enzyme perforin (**C**), on Vδ 2 T cells was detected by flow cytometry before and after one month HCV therapy. n = 7 for each group. (**D,E**) Expression of CD107a (**D**) and IFN-γ (**E**) on Vδ2 T cells before and after one month HCV therapy following zoledronate stimulations was also analyzed by flow cytometry. n = 7 for each group. (**F**) Supernatant levels of IFN-γ released in culture by Vδ2 T cells before and after one month HCV therapy after zoledronate stimulation were quantified by ELISA. Results (pg/ml of IFN-γ) were normalized for 1000 Vδ2 T cells. n = 7 for each group. *p < 0.05.

IFN-α has been used as an immunoregulatory and antiviral agent to treat chronic HCV infection^28^, we next evaluated *in vivo* effects of IFN-α administration during standard therapy on Vδ2 T -cell activity. Phenotypic and functional analysis of Vδ2 T cells was performed before and after one month IFN-α/RBV treatment in 7 chronic HCV-infected patients. The frequency of Vδ2 T cells was not significantly modified during IFN-α/RBV therapy (Fig. 8A). *In vivo* IFN-α treatment induced upregulation of the acivation markers CD38 and CD69 onVδ2 T cells (Fig. 8B). Vδ2 T-cell cytolytic enzymes perforin expression (Fig. 8C) and degranulation (Fig. 8D) was also greatly enhanced by IFN-α treatment. In stark contrast, flow cytometric and ELISA assays showed that IFN-γ production from Vδ2 T cells was further decreased following IFN-α therapy (Fig. 8E,F). Although we cannot completely rule out a possible effect of RBV on Vδ2 T cell activity, our *in vitro* experiment showed that RBV had little impact on Vδ2 T cell phenotype and function (Fig. S3A–D), suggesting that IFN-α play a major role in this effect. Thus, *in vivo* IFN-α administration further decreased Vδ2 T cell IFN-γ production and biased Vδ2 T cell function toward cytotoxicity in chronic HCV infections.

## Discussion

In humans, γδ T cells consist of 2 main subsets: Vδ1 T cells and Vδ2 T cells. Our present study mainly focused on Vδ2 T cells, however, Vδ1 T cells, which are the major γδ population in liver^7^, may also play an important role in chronic HCV infection. Tissue localization and as yet ill-defined functions of Vδ1 T cells limits their study in humans^7^,^29^. We observed no obvious correlation between peripheral Vδ1 T cells and viral loads or liver injury in patients infected with HCV, studies utilizing liver biopsy to analyze the exact role of Vδ1 T cells during chronic HCV infection are currently in progress in our laboratory.

In this paper, we analyzed and characterized Vδ2 T cells in patients with HCV infection. We first assessed the activation and differentiation profile of Vδ2 T cells and found that there was an increase in activated and effector Vδ2 T cells in patients, which is evidence that Vδ2 T cells are part of the active immune response against HCV. Also, the frequency of Vδ2 T-effector cells had a significant positive correlation with the level of liver injury marker ALT, suggesting a pathogenic role of Vδ2 T cells in HCV infection.

Cytolysis and IFN-γ production are two independent effector functions of activated Vδ2 T cells. Vδ2 T cell cytotoxic potential (direct cytotoxicity) appears to correlate with the expression of CD56 and CD16^23^,^24^. By use of CD56 and CD16 as a marker for Vδ2 T -cell cytotoxicity, we found that function in patients was higher than that in HCs, indicating acquisition of cytotoxic effector function by Vδ2 T cells during chronic HCV infection. CD56 + Vδ2 T cell frequencies in patients were shown to correlate positively with serum ALT levels. Meanwhile, cytolytic effector molecules of Vδ2 T cell such as perforin and granzyms were also clearly enhanced in HCV-infected patients with elevated ALT levels. These data suggest that Vδ2 T cell cytotoxic activity is closely associated with liver injury in patients. Surprisingly, IFN-γ production by Vδ2 T cells was not enhanced and was even lower in patients than in HCs. So, Vδ2 T cell activation during chronic HCV infection did not result in an equal increase of all effector functions. Vδ2 T cells seem to be impaired especially in their ability to secrete IFN-γ in HCV infection, thus, the cytototoxic potential of Vδ2 T cells prevails. Our findings support the concept that Vδ2 T cells *in vivo* are predominantly biased towards cytolytic activity in HCV-infected patients.

Chronic HCV infection is characterized by continuous liver inflammation and viral persistence. IFN-γ plays a critical role in anti-HCV infection and it has been demonstrated to be a powerful noncytolytic mechanism of viral clearance from infected hepatocytes^1^,^30^. We found that chronic HCV infection induced a severely impaired IFN-γ production from Vδ2 T cells, which may be an important mechanism contributing to HCV persistence. Overall, Vδ2 T cells from HCV-infected patients were of a predominantly activated/effector phenotype and characterized by a functional dichotomy, featuring an enhanced cytotoxicity coupled to reduced IFN-γ production. This may be explained that in the context of a chronic infection, such as HCV infection, persistent activation of Vδ2 T cells could drive their terminal differentiation and dysfunction. The dysfunction of Vδ2 T cells during chronic HCV infection may lead to chronic liver inflammation via cytotoxic mechanisms but not to HCV clearance because of insufficient IFN-γ production.

We then investigated the cause of functional changes in Vδ2 T cell during chronic HCV infection. HCV is a good inducer of type I IFN expression, possibly because it replicates via dsRNA intermediaries^27^. It has been reported that HCV-infected patients show persistent serum levels of IFN-α before therapy^26^. However, even though HCV replicons are highly sensitive to type I IFNs *in vitro*, HCV seems to be unresponsive to IFN α/β effects *in vivo*^31^. As a result, Vδ2 T cells in patients are exposed constantly to HCV-induced IFN-α, which may induce Vδ2 T cells function biased towards cytotoxicity. Indeed, *in vitro* exposure of Vδ2 T cells to IFN-α induced Vδ2 T cell activation and expression of cytolytic molecules without upregulating IFN-γ production. Thus, the functional dichotomy of Vδ2 T cells in HCV-infected patients could be recapitulated *in vitro* by exposure to IFN-α.

HCV eradication rates are limited to around 50% for patients who received therapy based on a combination of PEG-IFN-α and RBV^32^. Interestingly, we observed that *in vivo* IFN-α treatment after 1 month significantly decreased IFN-γ production from Vδ2 T cells. It is well established that effective T-cell response requires both an antigen-specific signal through the T cell antigen receptor (TCR) and an antigen-independent costimulatory signal^33^, and T-cell responsiveness was previously shown to be affected by repeated costimulation signals in the absence of a TCR-mediated antigenic signal^34^. We speculate that *in vivo* repeated IFN-α treatment without direct antigenic stimulation, as during standard therapy, induced a reduction of IFN-γ production from Vδ2 T cells. Likewise, in the context of chronic HCV infection, peripheral Vδ2 T cells also received persistent stimulation by serum IFN-α but without phosphoantigen stimulation, which may lead to their defective IFN-γ production. So, an inappropriate type I IFN induction during chronic HCV infection might cause defective IFN-γ production from Vδ2 T cells. The defective IFN-γ production from Vδ2 T cells in response to IFN-α might be one of the mechanisms which make some HCV-infected patients resistant to IFN-α based therapy.

Recently, several studies have demonstrated that NK cells, another important innate immune cell, were also characterized by a functional dichotomy in chronic viral hepatitis^35^,^36^,^37^. NK cells in the livers of HBV- infected patients were activated and skewed toward cytolytic activity but without a concomitant increase in IFN-γ production^38^. Similarly, NK cells of patients with chronic HCV infection displayed increased cytolytic activity but significantly impaired IFN-γ production^36^,^39^. These data, together with our result, suggest that functional dichotomy of immune cells such as NK cell and Vδ2 T cell may be a feature of chronic viral hepatitis. Vδ2 T cells have been shown to directly regulate NK cell-mediated antitumor cytotoxicity through CD137 engagement^40^, it will be interesting to determine whether Vδ2 T cells can directly activate NK cells and, if so, which molecular interactions are involved during chronic HCV infection.

Taken together, we have shown that Vδ2 T cells from patients with chronic HCV infection have an functional dichotomy phenotype, with an enhanced cytotoxicity and a reduced IFN-γ production, which are driven by chronic exposure to HCV induced IFN-α. The functional dichotomy would eventually lead to the liver damage without virus clearance seen in patients. This study extends our knowledge about the characteristics of Vδ2 T cells in HCV-infected patients and provides new insights that may be helpful in designing HCV immunotherapy.

## Materials and Methods

### Study population

Data reported in this study were obtained from 43 patients with chronic HCV infection, and 39 healthy age- and sex-matched volunteers as described in Supplementary Table S1. Patients were excluded if they were coinfected with HIV or other hepatitis viruses or had received antiviral or immunomodulatory HCV treatment before blood sampling. 7 chronic HCV-infected patients given an anti-viral combination treatment of pegylated interferon (Peg-IFN)-α (180μg/week, Pegasys; Roche) and Ribavirin (RBV) (1g/day, Copegus; Roche) were also enrolled. The study protocol was approved by the ethical committee of the Second Affiliated Hospital of Chongqing Medical University, and written informed consent was obtained from all participants. The methods were carried out in accordance with the approved guidelines.

Peripheral blood mononuclear cells (PBMCs) were isolated from all enrolled subjects by Ficoll-Isopaque (TBDscience, China) gradient centrifugation. Paired analysis of liver and blood Vδ2 T cells was performed for 8 patients. Except for the pathological evaluation, liver biopsy specimens were homogenized for the isolation of liver-infiltrating lymphocytes (LILs). The basic clinical data for the HCV-infected subjects with available liver biopsy samples are presented in Supplementary Table S2.

### Flow cytometric analysis

Human PBMCs or LILs were prepared and stained with conjugated monoclonal antibodies (mAbs). Abs against the following proteins were used: anti-CD3 (SK7), anti-CD16 (CB16), anti-CD56 (CMSSB), anti-CD38 (HB7), anti-CD69 (FN50), anti-FasL (NOK-1), anti-NKG2D (1D11), anti-CD107a (eBioH4A3), anti-perforin (dG9), anti-IFN-γ (4S.B3), mouse IgG1 (P3.6.2.8.1), mouse IgG2a (eBM2a), anti-mouse IgG2a (m2a-15F8) (eBioscience) and anti-TCRγ/δ (B1), anti-GranzymA(GrA) (CB9) (Biolegend) and anti-Vδ2 (B6), anti-Granulysin (RB1), anti-GranzymB(GrB) (GB11) (BD PharMingen) and anti-NKG2A (131411) (R&D) and anti-Vδ1 (TS8.2) (Thermo Scientific) and anti-IFNAR2 (MMHAR-2) (PBL Assay Science). Data were collected on a BD FACSCanto^TM^ II cytometer and analyzed using FlowJo analysis software 7.6.1 (Tristar).

### ELISA and intracellular cytokine analysis

PBMCs (10^6^/mL) were incubated with zoledronic acid (10 μM /ml, zoledronate, Sigma) in 200 μl RPMI 1640 medium, supplemented with 10% FBS for 24 hours. Supernatant levels of IFN-γ from culture system were determined using ELISA kits obtained from Dakewe Biotech Company. Results were normalized for 1,000 Vδ2 T cells.

For intracellular staining of IFN-γ, PBMCs or LILs were stimulated in complete RPMI 1640 medium with zoledronate (10 μM /ml) for 18 hours. Brefeldin A (BD PharMingen) was added 1hour after stimulation to block intracellular transport. Then the cells were harvested and stained following standard procedures.

### CD107a degranulation assay

PBMCs or LILs were cultured with zoledronate (10 μM /ml) and anti-CD107a in complete RPMI 1640 for 18 hours. Mononesin (eBioscience) were directly added into the medium 1 h after stimulation. Then cells were collected and stained with surface antibodies for ordinary FACS staining.

### Vδ2 T cell purification

Vδ2 T cells were purified through positive selection by using anti-Vδ2 phycoerythrin (PE) and anti-PE microbeads (Miltenyi Biotech, Bergisch Gladbach, Germany). The purity of Vδ2 T cells subset was >90%.

### IFN-α stimulation

PBMCs were preincubated at 37 °C in complete medium with or without 100 U/mL IFN-α (R&D) for 24 hours. PBMCs then either were stained directly with antibodies for flow cytometric analysis, or used for degranulation assays and cytokine release assays as described earlier.

### Ribavirin stimulation

PBMCs were preincubated at 37 °C in complete medium with or without 5 μg/mL ribavirin (RBV, Sigma) for 24 hours. PBMCs then either were stained directly with antibodies for flow cytometric analysis, or used for degranulation assays and cytokine release assays as described earlier.

### Generation of Vδ2T cell lines and cytotoxicity assay

For Vδ2 T cell expansion, PBMCs (10^6^/mL) were cultured with complete RPMI 1640 supplemented with 10% FBS in 24 well plates and stimulated with IL-2 (100 U/ml) in combination with zoledronate (5 μM /ml). Zoledronate were added as an initial single dose, fresh IL-2 was added every 3 days. Cells were analyzed by flow cytometry at day 10. At this time point, about 70–90% of lymphocytes were Vδ2 T cells. From these cells, Vδ2 T cells were isolated by positive selection with magnetic activated cell sorting (MACS) system. Cytotoxicity assays were performed using the CytoTox 96 nonradioactive cytotoxicity assay (Promega Corp, Madison WI, USA), according to the manufacturer’s protocol. Vδ2 T cells were preincubated with or without 100 U/mL IFN-α for 24 hours and were subsequently incubated with K562 or HepG2 cells at the ratios of 20:1 for 6 hours. The maximum and spontaneous release was determined by incubating the target cells and effector cells with 0.5% Triton X-100 or medium alone, respectively. Percent cytotoxicity was calculated by the following formula:


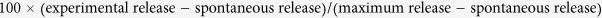


### Statistical analysis

The Mann-Whitney *U* test was used to compare data between HCV-infected patients and HCs. Paired Student *t* tests were used to assess differences in Vδ2 T cell phenotypes between paired liver and blood samples, and to assess changes in Vδ2 T cell phenotypes on *in vitro* or *in vivo* stimulation with IFN-α. Correlations between variables were evaluated with the Spearman rank correlation test. *P* <* *0.05 was considered statistically significant for all tests. Calculations were performed using GraphPad Prism version 4.00 (GraphPad Software, Inc., San Diego).

## Additional Information

**How to cite this article**: Yin, W. *et al*. Functional dichotomy of Vδ2 γδ T cells in chronic hepatitis C virus infections: role in cytotoxicity but not for IFN-γ production. *Sci. Rep.* **6**, 26296; doi: 10.1038/srep26296 (2016).

## Supplementary Material

Supplementary Information
