# Supplementary material for: Functional dichotomy of Vδ2 γδ T cells in chronic hepatitis C virus infections: role in cytotoxicity but not for IFN-γ production
Source: Sci Rep. 2016 May 19;6:26296. doi: 10.1038/srep26296 (PMC4872040; doi:10.1038/srep26296)
Supplement: Supplementary Information [file srep26296-s1.doc]

**Functional dichotomy of Vδ2 γδ T cells in chronic hepatitis C virus infections: role in cytotoxicity but not for IFN-γ production**

Wenwei Yin, Shiwen Tong, Qiongfang Zhang, Jianying shao, Qian Liu, Hong Peng, Huaidong Hu, Mingli Peng, Peng Hu, Hong Ren, Zhigang Tian, and Dazhi Zhang

**Supplementary Information**

**Supplementary TableS1. Clinical characteristics of Healthy Controls and HCV-infected patients enrolled in this study**

|  | **Healthy Controls**  **n=39** | **HCV-infected Patients**  **n=43** |
| --- | --- | --- |
| Gender (Female: Male) | 18:21 | 20:23 |
| Age, years: median (range) | 31(18-58) | 35(21-54) |
| ALT, IU/L: median(range) | ＜40 | 103(23-293) |
| AST,IU/L: median(range) | ＜34 | 69.5(23-172) |
| HCV RNA, log10 copies/ml:  median (range) | nt | 6.377(3.87-6.859) |

nt: not tested.

**Supplementary Table S2. Patient characteristics with available liver biopsy specimens.**

| Gender | Age | HCV genotype | HCV RNA, *IU/mL* | ALT level, *U/mL* | Ishak inflammatory score | Ishak fibrosis score |
| --- | --- | --- | --- | --- | --- | --- |
| F | 39 | 3 | 40,500 | 27 | 4 | 3 |
| F | 51 | 3 | 2,590,000 | 92 | 6 | 0 |
| F | 34 | 3 | 5,020,000 | 56 | 7 | 0 |
| F | 40 | 3 | 6,140,000 | 45 | 7 | 0 |
| M | 42 | 3 | 14,100 | 149 | 8 | 3 |
| M | 35 | 3 | 519,000 | 103 | 5 | 2 |
| M | 43 | 1 | 2,690,000 | 182 | 9 | 1 |
| F | 46 | 1 | 428,000 | 102 | 6 | 1 |

**
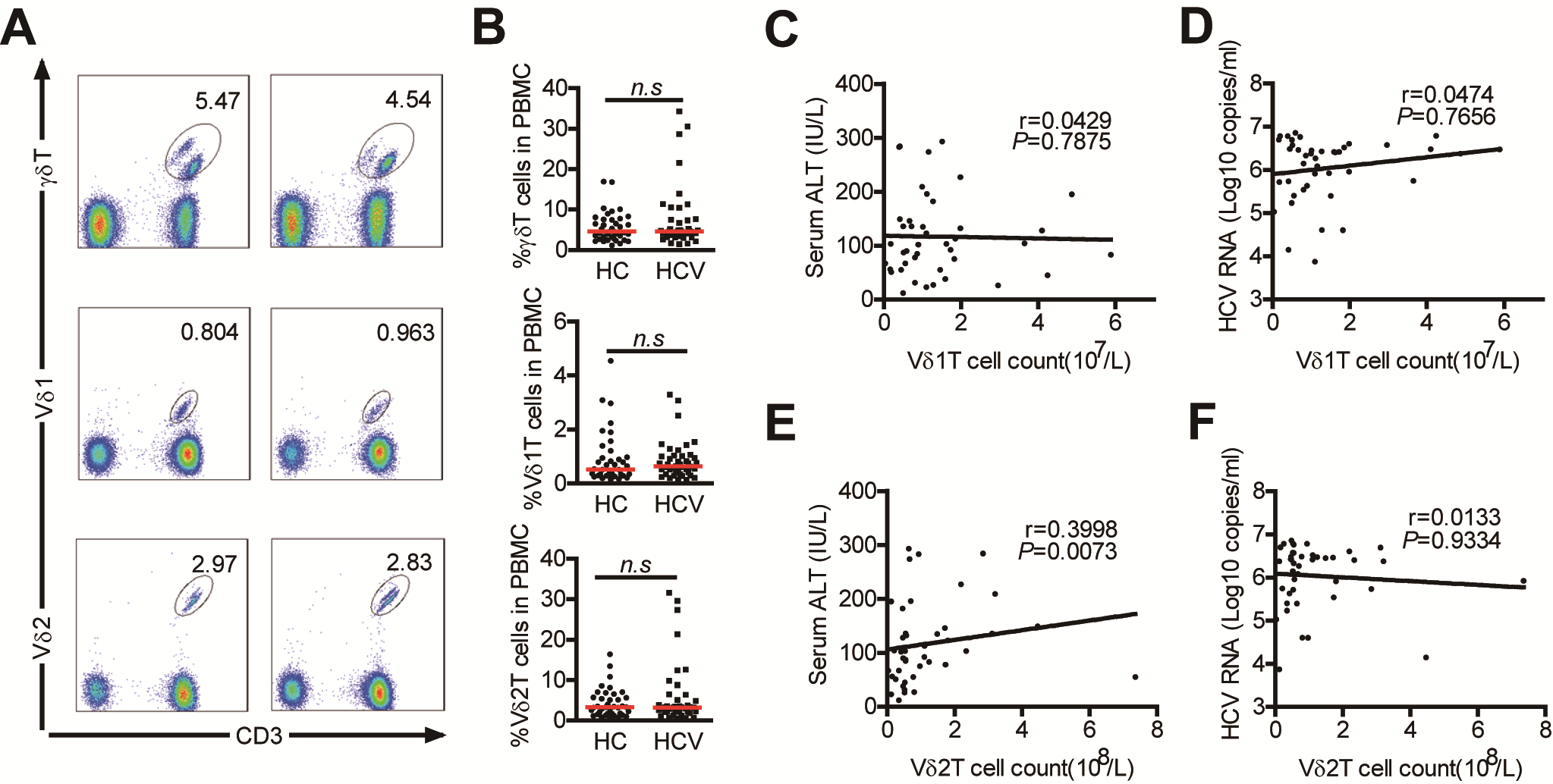
**

**Fig. S1. The number of circulating Vδ2 T cells is associated with liver damage in HCV-infected Patients.**

(A) Representative dot plots of peripheral blood γδT, Vδ2 T and Vδ1 T cells from HCs and HCV-infected patients. (B) Percentages of total γδ, Vδ2, and Vδ1 T cells in peripheral blood of HCs and HCV-infected patients. n = 39 and 43 for HC and HCV, respectively. Horizontal lines indicate the median. (C and D) Correlation analysis of the number of Vδ1 T cells and the serum ALT levels (C) or HCV RNA loads (D) in HCV-infected patients. n=43. (E and F) Correlation analysis of the number of Vδ2 T cells and the serum ALT levels (E) or HCV RNA loads (F) in HCV-infected patients. n=43.

**
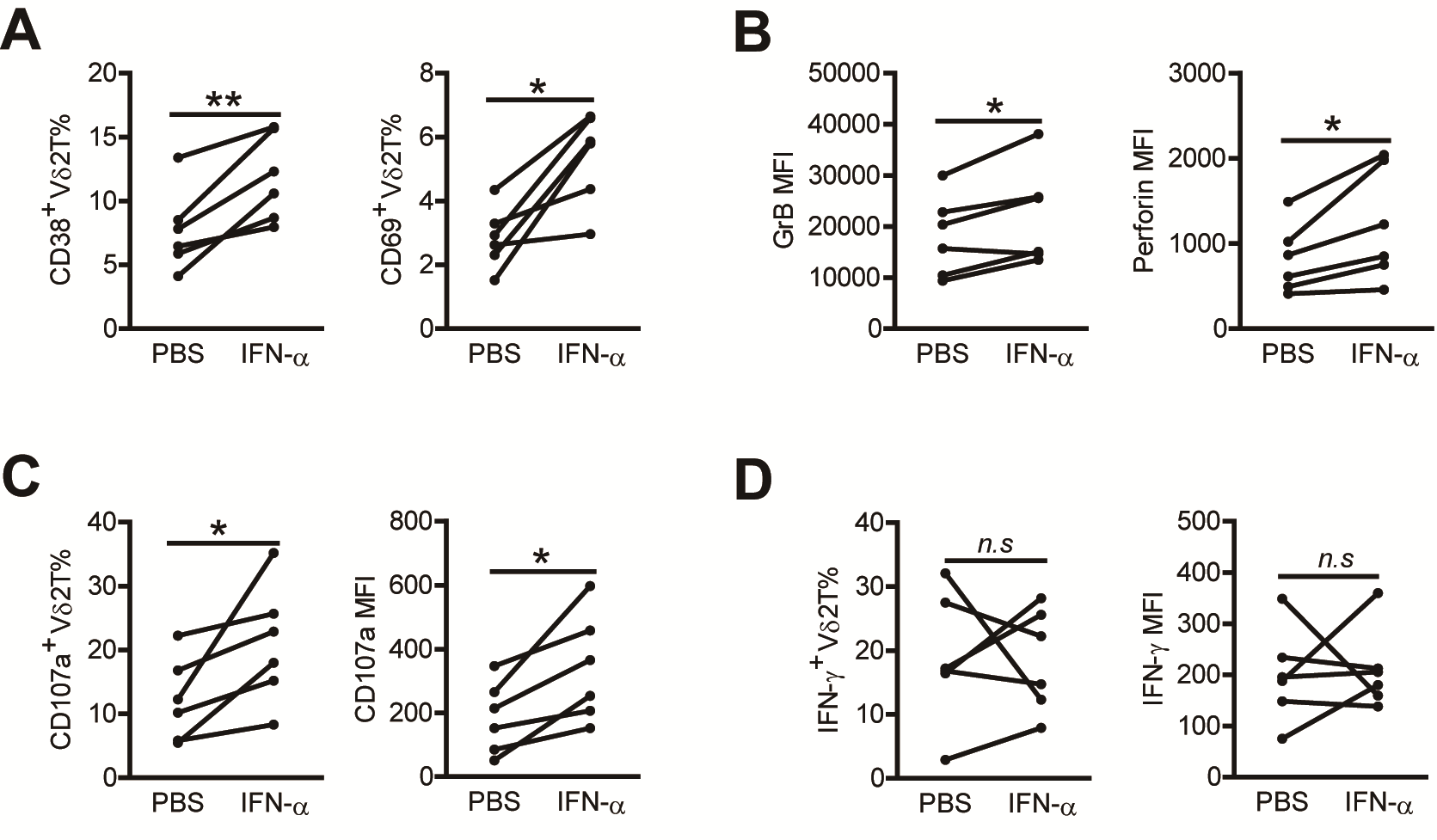
**

**Fig. S2. Vδ2 T cells of chronic HCV-infected patients are activated and upregulate cytolytic activity but not IFN-γ production after *in vitro* exposure to IFN-α.**

PBMCs from HCV-infected patients were preincubated with or without IFN-α for 24 h. (A,B) Expression of activation markers CD38 and CD69 (A), and cytolytic enzymes GrB and perforin (B) on Vδ2 T cells was assessed by flow cytometry. (C, D) Expression of CD107a(C) and IFN-γ (D) on Vδ2 T cells following zoledronate stimulations was analyzed by flow cytometry. n = 6 for each group. **p* < 0.05, ***p* < 0.01.

**
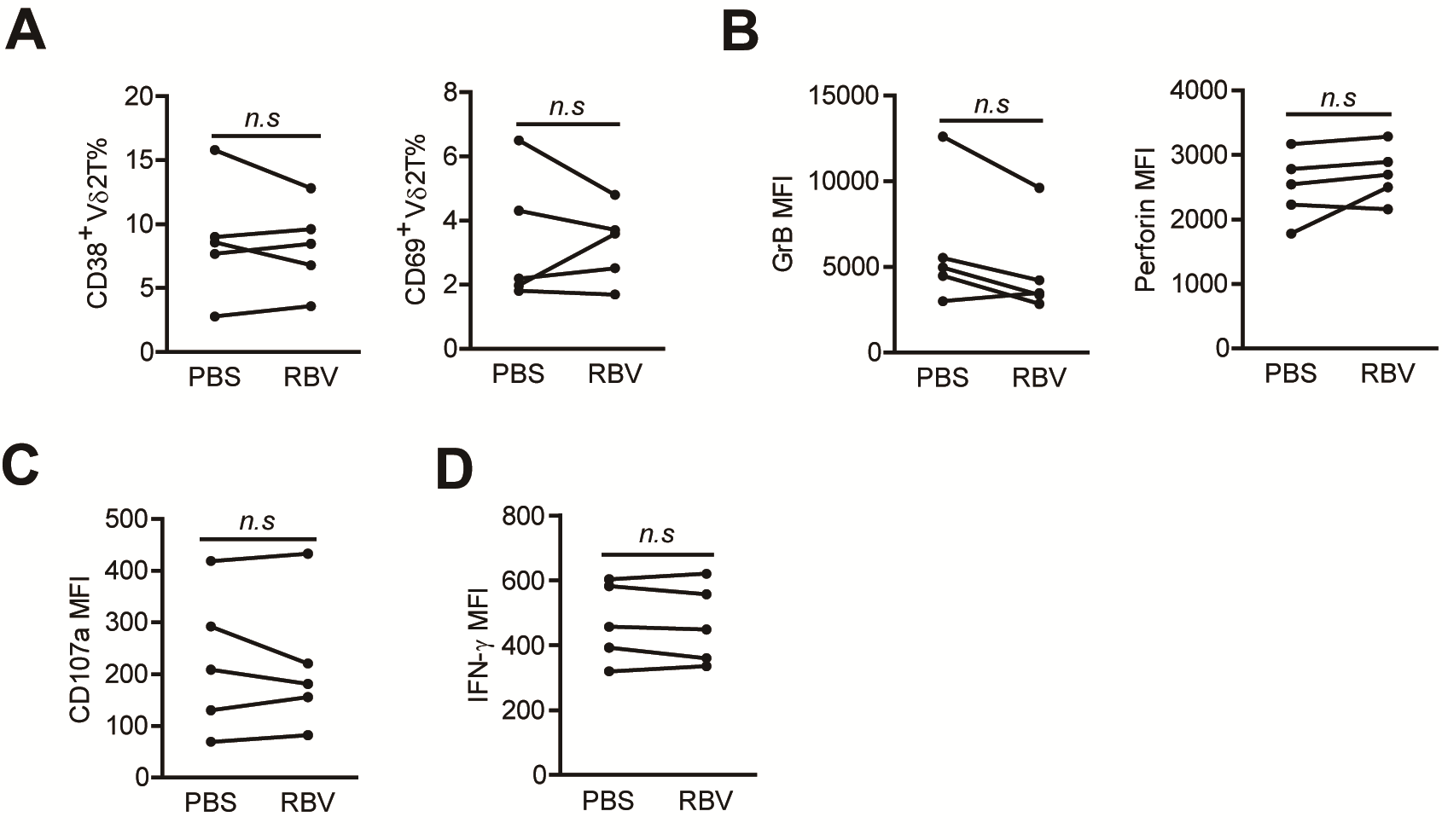
**

**Fig. S3. RBV has no effect on Vδ2 T cell phenotype and function.**

PBMCs from HCs were preincubated with or without RBV for 24 h. (A,B) Expression of activation markers CD38 and CD69 (A), and cytolytic enzymes GrB and perforin (B) on Vδ2 T cells was assessed by flow cytometry. (C, D) Expression of CD107a (C) and IFN-γ (D) on Vδ2 T cells upon zoledronate stimulation was analyzed by flow cytometry. n = 5 for each group.
